# Prognostic significance of molecular subgroups in survival outcome for children with medulloblastoma in Malaysia

**DOI:** 10.3389/fonc.2023.1278611

**Published:** 2023-10-18

**Authors:** Revathi Rajagopal, Ay Jiuan Teng, Vida Jawin, Oy Leng Wong, Hakimah Mahsin, Nor Haizura Abd Rani, Tsiao Yi Yap, Kogilavani Gunasagaran, Asohan Thevarajah, Seoh Leng Yeoh, Gek Bee Ong, Hany Ariffin, David Jones, Eric Bouffet, Nicholas G. Gottardo

**Affiliations:** ^1^Division of Hematology, Oncology and Bone Marrow Transplantation, Department of Pediatrics, University Malaya Medical Center, Kuala Lumpur, Malaysia; ^2^Division of Hematology and Oncology, Department of Pediatrics, Sarawak General Hospital, Ministry of Health Malaysia, Kuching, Malaysia; ^3^Division of Hematology and Oncology, Department of Pediatrics, Hospital Sultan Ismail, Ministry of Health Malaysia, Johor Bharu, Malaysia; ^4^Department of Pathology, Sarawak General Hospital, Ministry of Health Malaysia, Kuching, Malaysia; ^5^Department of Pathology, Penang General Hospital, Ministry of Health Malaysia, George Town, Malaysia; ^6^Department of Pathology, Queen Elizabeth Hospital, Ministry of Health Malaysia, Kota Kinabalu, Malaysia; ^7^Division of Hematology and Oncology, Department of Pediatrics, Sabah Woman and Children’s Hospital, Ministry of Health, Kota Kinabalu, Malaysia; ^8^Division of Hematology and Oncology, Department of Pediatrics, Penang General Hospital, Ministry of Health Malaysia, George Town, Malaysia; ^9^Division of Pediatric Neuro-oncology, German Cancer Research Centre, Heidelberg, Germany; ^10^Division of Neuro-oncology, Department of Pediatric Hematology and Oncology, Hospital for Sick Children, Toronto, Ontario, Canada; ^11^Department of Pediatric and Adolescent Oncology/Hematology, Perth Children’s Hospital, Nedlands, Perth, WA, Australia; ^12^Brain Tumor Research Program, Telethon Kids Institute University of Western Australia, Nedlands, Perth, WA, Australia

**Keywords:** survival outcome, medulloblastoma, Wingless, Sonic Hedgehog, Group 3, Group 4, abandonment

## Abstract

**Introduction:**

Advancements in genomic profiling led to the discovery of four major molecular subgroups in medulloblastoma (MB), which have now been incorporated into the World Health Organization classification of central nervous system tumors. The current study aimed to determine the prognostic significance of the MB molecular subgroups among children in Malaysia.

**Methods:**

We assembled MB samples from children <18 years between January 2003 and June 2017 from four pediatric oncology centers in Malaysia. MB was sub-grouped using 850k DNA methylation testing at German Cancer Research Centre, Heidelberg, Germany.

**Results:**

Fifty samples from patients diagnosed and treated as MB were identified. Two (4%) of the 50 patients’ tumor DNA samples were insufficient for analysis. Of the remaining 48 patients, 41 (85%) samples were confirmed as MB, while for 7 (15%) patients, DNA methylation classification results were discrepant with the histopathological diagnosis of MB, with various other diagnoses. Of the 41 MB patients, 15 patients were stratified as standard-risk (SR), 16 patients as high-risk (HR), and ten as infants (age <3 years old). Molecular subgrouping of the whole cohort revealed four (14%) WNT, 11 (27%) SHH, 10 (24%) Group 3, and 16 (39%) Group 4. Treatment abandonment rates for older children and infants were 22.5% and 10%, respectively. After censoring treatment abandonment, for SR patients, the 5-year event-free survival (EFS) and overall survival (OS) were 43.1% ± 14.7% and 46.9 ± 15.6%, respectively, while in HR, 5-year EFS and OS were both 63.6% ± 14.5%. Infants had a 5-year EFS and OS of 55.6% ± 16.6% and 66.7% ± 15.7%, respectively. WNT tumors had the best 5y-OS, followed by Group 3, Group 4, and SHH in children ≥3 years old. In younger children, SHH MB patients showed favorable outcomes.

**Conclusion:**

The study highlights the importance of DNA methylation profiling for diagnostic accuracy. Most infants had SHH MB, and their EFS and OS were comparable to those reported in high-income countries. Due to the relatively small cohort and the high treatment abandonment rate, definite conclusions cannot be made regarding the prognostic significance of molecular subgroups of MB. Implementing this high-technology investigation would assist pathologists in improving the diagnosis and provide molecular subgrouping of MB, permitting subgroup-specific therapies.

## Introduction

Medulloblastoma (MB), the most common malignant central nervous system (CNS) tumor of childhood, demonstrates high biological and clinical heterogeneity (1, 2). Historically, MB risk stratification was based on age, extent of surgical resection, residual tumor, metastatic status, and histological subtype (3–5). MB was originally classified into four histologic variants predominantly based on features seen on light microscopy and conventional histological stains. These variants were medulloblastoma with extensive nodularity (MBEN), desmoplastic-nodular (DN), classic, and large-cell–anaplastic (LCA).

The standard of care for MB for children ≥3 years old consists of maximal surgical resection, risk-adapted craniospinal irradiation (CSI), and adjuvant chemotherapy. Standard-risk (SR) MB is defined by complete or near total resection with residual tumor < 1.5cm^2^ and absence of metastatic disease. Patients with post-surgical residual tumor > 1.5cm^2^, metastatic dissemination, and LCA histology in some studies were classified as having high-risk (HR) MB (5). SR MB patients receive CSI of 23.4Gy with a boost up to 54-55Gy to the posterior fossa or tumor bed, followed by adjuvant chemotherapy. Whilst HR MB patients are treated with a higher CSI dose of 36-39Gy with a boost up to 54-55Gy to the posterior fossa or tumor bed, followed by adjuvant chemotherapy (5, 6). Using these approaches, the 5-year overall survival (OS) rate in high-income countries is approximately greater than 80% in SR MB patients and 53-76% in HR MB patients (6–9). For children <3 years old, radiotherapy-sparing approaches have become the accepted standard and the survival outcomes vary based on histology subclass, post-operative residual tumor, and extent of metastasis (10–12). In recent trials conducted in Europe and North America, the 5-year OS rates in children <3 years old who had complete resection, residual tumor, and metastases were 79-93%, 57%, and 38%, respectively (10–12). Based on histology, young children with MBEN/DN, classic, and LCA histologies showed 5-year OS rates of 78-100%, 41-67%, and 33%, respectively (10–12).

Over the past 15 years, through marked advances in genomic studies, our understanding of MB biology has dramatically evolved, culminating in four core distinct molecular subgroups termed: Wingless (WNT), Sonic Hedgehog (SHH), Group 3 (G3), and Group 4 (G4) (1, 13). These molecular subgroups display different genetic, clinical characteristics, recurrence patterns, and survival outcomes (14–17). These subgroups were incorporated into the revised WHO 2016 classification and integrated with the histological variants for improved classification and prognostic correlation (13). In children, G4 is the most frequent MB subgroup representing 40-45% of all MBs, followed by SHH (28-30%), G3 (25-28%), and WNT (10-15%) (2, 18). WNT subgroup patients have an excellent prognosis whilst G3 patients demonstrate worse outcomes (17).

Methylation of the cytosine component of DNA in cytosine-phosphate-guanine (CpG) dinucleotides is a crucial biological mechanism in determining gene expression. Cancers have complex methylation profiles, thus DNA methylation signatures based on thousands of CpG sites can provide robust data for precise diagnosis even when not all histological or molecular features of a tumor are detected. DNA methylation profiling is now considered the gold standard for MB subgrouping due to its unbiased method (19). The German Cancer Research Centre (DKFZ) developed DNA methylation-based CNS tumor classification using a comprehensive machine learning approach to improve the diagnostic accuracy of the clinical decision-making process. This method has been shown to be highly robust and reproducible with a high level of standardization. It reduces the inter-observer variability even from a small sample and poor-quality material (19).

To date, limited data have been reported from low and middle-income countries (LMIC) on pediatric MB patients in relation to the four molecular subgroups (20–22). Indeed, no data exist from Malaysia. Therefore, we performed a retrospective study of molecular classification of pediatric MB to investigate the subgroup-specific percentage and survival outcomes from the four tertiary pediatric oncology centers in Malaysia using 850k DNA methylation profiling. In addition, we compared the accuracy of histological diagnosis with immunohistochemistry (IHC) and DNA methylation profiling on the diagnostic tumor tissue.

## Patients and methods

Children ≤ 18 years old diagnosed with MB at University Malaya Medical Center (UMMC), Penang General Hospital (PGH), Sarawak General Hospital (SGH), and Sabah Women and Children’s Hospital (SWCH), Malaysia between January 2003 and June 2017 were reviewed. Archived formalin-fixed paraffin-embedded (FFPE) tumor tissues from these patients were retrieved from the respective pathology departments. The retrieved samples were sent to DKFZ for MB molecular subgroup analysis using the 850k DNA methylation array technique. Clinical data were collected from medical charts, radiological results, and follow-up clinic records. These children were followed up until November 2020 to evaluate the survival outcome.

### Statistical analysis

Event-free survival (EFS) was measured from the date of diagnosis to the date of disease recurrence, death, or last follow-up. OS was measured from the date of diagnosis to the date of death or last follow-up. Survival curves were constructed using Kaplan-Meier methods. Statistical significance was defined as a p-value < 0.05. Data analysis was performed using the software IBM SPSS Statistics 27 (IBM Corp., Armonk, NY, USA).

### Ethics statement

This study was approved by the Ministry of Health (MOH) Medical Research and Ethics Committee (NMRR-17-991-35677) and UMMC Medical Research Ethics Committee (MREC-2016112-4485).

## Results

### Comparison between histological diagnosis and 850k DNA methylation profiling results of the whole cohort

A total of 50 samples derived from patients diagnosed and treated as MB were identified. The histological diagnosis and molecular subgrouping were analyzed with 850k DNA methylation profiling. Two (4%) of the 50 patients’ tumor DNA samples were insufficient for analysis. Of the remaining 48 patients, 41 (85%) samples were confirmed as MB, whilst for seven (15%) patients, DNA methylation classification results were discrepant with the histopathological diagnosis of MB. These included glioblastoma multiforme (GBM) (n =2), atypical teratoid rhabdoid tumor (n=2), and one each of Ewing sarcoma, malignant peripheral nerve sheath tumor (MPNST) like sarcoma, and pineoblastoma. All seven patients received MB therapy, and six of them died due to progressive disease, except one patient with Ewing Sarcoma survived despite receiving MB treatment (**Table 1**).

**Table:** Table 1 Demographic, diagnosis, treatment, and outcome.

Molecular subgroup	Mt	Surgery	Histology diagnosis from local hospitals	Histology subtypes from local hospitals	DNA methylation result from DKFZ	*MYCC/* *MYCN* amp. result from DKFZ	Upfront RT	CTX	Site of relapse	Time to relapse from diagnosis (years)	Outcome (Time from diagnosis to last follow-up/death in years)
Infants and young children (<3 years old)											
**SHH-INF (NOS-PQs)**	**0**	**STR**	**MB**	**Desmo-plastic**	**MB**	**N**	**N**	**HS II**	**N**	**N**	**Alive (7.75)**
**SHH-INF (type 2)**	**0**	**STR**	**MB**	**NOS**	**MB**	**N**	**N**	**POG Baby brain protocol**	**Primary site**	**2.58**	**Alive (5.83)**
**SHH-INF (type 2)**	**NI**	**STR**	**MB**	**MBEN**	**MB**	**N**	**N**	**Refused CTX**	**NI**	**NI**	**NI (0.33)**
**SHH-INF (type 1)**	**0**	**STR**	**MB**	**NOS**	**MB**	**N**	**N**	**POG Baby Brain protocol**	**PD-Primary site**	**1.66**	**Dead (1.66)**
**SHH-INF (type 1)**	**0**	**STR**	**MB**	**Desmo-plastic**	**MB**	**N**	**N**	**HS II**	**N**	**N**	**Alive (3.4)**
**SHH-INF (type 3)**	**0**	**NTR**	**MB**	**MBEN**	**MB**	**N**	**N**	**ACNS 1221**	**N**	**N**	**Alive (3.66)**
**SHH-INF (type 2)**	**0**	**STR**	**MB**	**Desmo-plastic**	**MB**	**N**	**N**	**ACNS 1221**	**N**	**N**	**Alive (3.25)**
**G3**	**0**	**NTR**	**MB**	**Classic**	**MB**	**N**	**N**	**HS II**	**N**	**N**	**Alive (6.25)**
**G3**	**3**	**NTR**	**MB**	**Classic**	**MB**	***MYCC***	**N**	**HS II**	**Spinal metastasis**	**1.1**	**Dead (2.0)**
**G3**	**0**	**NTR**	**MB**	**Classic**	**MB**	***MYCC***	**N**	**POG Baby Brain protocol**	**PD-Primary site, intracranial leptomeningeal and spine**	**0.51**	**Dead (0.66)**
Standard-risk ≥ 3 years old)											
**SHH-AD (type 4)**	**0**	**GTR**	**MB**	**Desmo-plastic**	**MB**	**N**	**CSI 36Gy, PSB 54Gy**	**Defaulted after 4 courses of CCNU, Cis, VCR**	**NI**	**NI**	**NI (1.67)**
**SHH-AD (type 3)**	**0**	**NTR** **<1.5cm^2^**	**MB**	**NOS**	**MB**	**N**	**CSI 23.4Gy, PSB 56Gy**	**Recurrence after 4 courses of CCNU/Cis/VCR**	**Primary site**	**1.16**	**Alive (4.83)**
**G3**	**0**	**NTR** **<1.5cm^2^**	**MB**	**NOS**	**MB**	**N**	**CSI 36Gy, PSB 54Gy**	**8 courses of CCNU, Cis, VCR**	**N**	**N**	**Alive (6.25)**
**G3**	**0**	**GTR**	**MB**	**NOS**	**MB**	**N**	**No RT** **ф physician decision**	**No CTX** **ф physician decision**	**Primary site and spinal metastasis**	**0.33**	**Dead (0.33)**
**G3**	**0**	**GTR**	**MB**	**Classic**	**MB**	***MYCC***	**CSI 23.4Gy,** **PSB 54Gy**	**8 courses of Cis, VCR, Cyclo**	**Right frontal lobe**	**2.25**	**Dead (4.0)**
**G3**	**0**	**GTR**	**MB**	**NOS**	**MB**	**N**	**CSI 36Gy, PSB 54Gy**	**8 courses of CCNU, Cis, VCR**	**N**	**N**	**Alive (4.11)**
**G4**	**0**	**GTR**	**MB**	**NOS**	**MB**	**N**	**Incomplete RT**	**N**	**N**	**N**	**Dead (0.5)** **Surgical infection during RT**
**G4**	**0**	**GTR**	**MB**	**Classic**	**MB**	***MYCN***	**CSI 36Gy, PSB 54Gy**	**8 courses of CCNU, Cis, VCR**	**N**	**N**	**Alive (14.16)**
**G4**	**0**	**GTR**	**MB**	**Desmo-plastic**	**MB**	**N**	**CSI 36Gy, PSB 54Gy**	**8 courses of CCNU, Cis, VCR**	*Primary site	**4.41**	**Dead (4.9)**
**G4**	**0**	**NTR** **<1.5cm^2^**	**MB**	**Classic**	**MB**	**N**	**CSI 36Gy, PSB 54Gy**	**Refused CTX**	**Primary site and spine**	**2.66**	**Dead (3.5)**
**G4**	**0**	**NTR**	**MB**	**NOS**	**MB**	**N**	**CSI 36Gy, PSB 54Gy**	**8 courses of CCNU, Cis, VCR**	N	**N**	**Alive (9.83)**
**G4**	**0**	**GTR**	**MB**	**NOS**	**MB**	**N**	**CSI 36Gy, PSB 54Gy**	**8 courses of CCNU, Cis, VCR**	**N**	**N**	**Alive (6.0)**
**G4**	**0**	**GTR**	**MB**	**NOS**	**MB**	**N**	**CSI 36Gy, PSB 54Gy**	**8 courses of CCNU, Cis, VCR**	**N**	**N**	**Alive (3.83)**
**G4**	**0**	**GTR**	**MB**	**NOS**	**MB**	**N**	**CSI 36Gy, PSB 54Gy**	**8 courses of CCNU, Cis, VCR**	**Primary site and spinal metastasis**	**3.16**	**Dead (4.25)**
**G4**	**0**	**GTR**	**MB**	**NOS**	**MB**	**N**	**CSI 36Gy, PSB 54Gy**	**4 courses of CCNU, Cis, VCR**	**N**	**0.91**	**Dead (0.91)** **Sepsis after chemotherapy**
High-risk (≥ 3 years old)											
**WNT**	**0**	**NTR >1.5cm^2^**	**MB**	**Classic**	**MB**	**N**	**CSI 36Gy, PSB 54Gy**	**8 courses of CCNU, Cis, VCR**	**N**	**N**	**Alive (6.67)**
**WNT**	**0**	**STR**	**MB**	**Desmo-plastic**	**MB**	**N**	**Refused RT**	**Refused CTX**	**NI**	**NI**	**NI (0.04)**
**WNT**	**NI**	**GTR**	**MB**	**NOS**	**MB**	**N**	**Refused RT**	**Refused CTX**	**Primary site**	**0.33**	**Dead (0.66)**
**WNT**	**3**	**GTR**	**MB**	**NOS**	**MB**	**N**	**CSI 36Gy, PSB 54Gy**	**8 courses of CCNU, Cis, VCR**	**N**	**N**	**Alive (5.50)**
**SHH-INF (NOS-PQs)**	**3**	**GTR**	**MB**	**Classic**	**MB**	**N**	**CSI 36Gy, PSB 54Gy**	**4 courses of CCNU, Cis, VCR**	*Primary site	**0.83**	**Dead (0.83)**
**SHH-AD (type 3)**	**0**	**STR**	**MB**	**NOS**	**MB**	**N**	**CSI 36Gy, PSB 54Gy**	**8 courses of CCNU, Cis, VCR**	**Primary site**	**1.42**	**Death (1.5)**
**G3**	**3**	**NTR >1.5cm^2^**	**MB**	**NOS**	**MB**	***MYCC***	**Refused RT**	**HS II (1 course then defaulted)**	**PD-primary site**	**0.75**	**Dead (0.75)**
**G3**	**NI**	**GTR**	**MB**	**NOS**	**MB**	**N**	**Refused RT**	**Refused CTX**	**NI**	**NI**	**NI (0.25)**
**G3**	**3**	**NTR** **>1.5cm^2^**	**MB**	**NOS**	**MB**	**N**	**CSI 36Gy, PSB 54Gy**	**8 courses of CCNU, Cis, VCR**	**N**	**N**	**Alive (4.0)**
**G4**	**1**	**GTR**	**MB**	**Classic**	**MB**	**N**	**CSI 36Gy, PSB 54Gy**	**8 courses of CCNU, Cis, VCR**	**Primary site**	**1.75**	**Dead (2.0)**
**G4**	**2**	**STR**	**MB**	**NOS**	**MB**	**N**	**CSI 36Gy, PSB 54Gy**	**8 courses of CCNU, Cis, VCR**	**N**	**N**	**Alive (7.13)**
**G4**	**3**	**NTR >1.5cm^2^**	**MB**	**NOS**	**MB**	***MYCN***	**CSI 45Gy, PSB 54Gy**	**8 courses of CCNU, Cis, VCR**	**N**	**N**	**Alive (6.58)**
**G4**	**0**	**NI**	**MB**	**NOS**	**MB**	**N**	**Refused RT**	**Refused CTX**	**NI**	**NI**	**NI (0.08)**
**G4**	**3**	**GTR**	**MB**	**NOS**	**MB**	**N**	**CSI 36Gy, PSB 54 Gy, spine T2-T9 54Gy**	**8 courses of CCNU, Cis, VCR**	**N**	**N**	**Alive (5.0)**
**G4**	**3**	**GTR**	**MB**	**NOS**	**MB**	***MYCC***	**CSI 36Gy, PSB 54Gy**	**1 course of CCNU, Cis, VCR**	*Third ventricle	**0.75**	**Dead (0.83)**
**G4**	**3**	**GTR**	**MB**	**Classic**	**MB**	**N**	**CSI 39.6 Gy, PSB 54Gy**	**POG 9031 (3 courses of Cis/Eto & 7 courses of Cyclo/VCR)**	**N**	**N**	**Alive (4.0)**
Discrepancy between local histopathological diagnosis and DNA methylation profiling results											
**-**	**0**	**NTR**	**MB**	**NOS**	**Sarcoma/** **MPNST like**	**-**	**CSI 36Gy,** **PSB 54Gy**	**Defaulted after 1 course of CCNU, Cis, VCR**	**Primary site**	**10.0**	**Dead (10.5)**
**-**	**0**	**Biopsy**	**MB**	**NOS**	**GBM**	**-**	**CSI 36Gy, PSB 54Gy**	**N**	**PD**	**0.83**	**Dead (1.0)**
**-**	**4**	**STR**	**MB**	**Classic**	**Pineo-blastoma**	**-**	**CSI 36Gy, PSB 54Gy**	**8 courses of CCNU, Cis, VCR**	**Primary site**	**1.5**	**Dead (1.66)**
**-**	**0**	**GTR**	**MB**	**NOS**	**Ewing Sarcoma**	**-**	**CSI 36Gy, PSB 54Gy**	**8 courses of CCNU, Cis, VCR**	**N**	**N**	**Alive (10.5)**
**-**	**0**	**STR**	**MB**	**NOS**	**ATRT**	**-**	**N**	**Baby brain protocol**	**PD**	**0.51**	**Dead (0.51)**
**-**	**0**	**STR**	**MB**	**NOS**	**GBM**	**-**	**CSI 36Gy, PSB 54Gy**	**PD after 1 course of Cis, CCNU, VCR**	**PD**	**0.58**	**Dead (1.0)**
**-**	**0**	**STR**	**MB**	**NOS**	**ATRT**	**-**	**N**	**Refused CTX**	**PD**	**0.5**	**Dead (0.5)**
Samples with insufficient tissue for DNA methylation profiling	**0**	**STR**	**MB**	**NOS**	**Normal tissue**	**-**	**N**	**N**	**N**	**N**	**Dead (0.25)** **Post-operative complication**
	**0**	**STR**	**MB**	**Classic**	**Insufficient** **tissue**	**-**	**CSI 36Gy, PSB 54Gy**	**8 courses of CCNU, Cis, VCR**	**N**	**N**	**Alive (9.25)**

amp, amplification; AD, adult; amp, amplification; ATRT, atypical teratoid rhabdoid tumor; CCNU, lomustine; Cis, cisplatin; CSI, craniospinal radiotherapy; CTX, chemotherapy; Cyclo, cyclophosphamide; DKFZ, German Cancer Research Center; Eto, etoposide; G3, Group 3; G4, Group 4; GTR, gross total resection; Gy, Gray; HR, high risk; HS, Headstart; Ifos, Ifosfamide; iHR, young children with high risk group; INF, infant; MB, medulloblastoma; Mt, metastasis; MPNST, malignant peripheral nerve sheath tumor; N, no; NI, no information; NTR, near total resection; NOS, not otherwise specified; PD, progressive disease; POG, Pediatric Oncology Group; PQs, poor quality sample; PSB, primary site boost; RT, radiotherapy; SHH, Sonic hedgehog; SR, standard-risk; STR, subtotal resection; TB, tumour bed; VCR, vincristine; WNT, Wingless.

* Spinal magnetic resonance imaging was not performed at relapse/disease progression.

Ф Physician’s decision for conservative treatment after surgery due to poor neurological status.

Color shading signifies certain molecular subgroup in medulloblastoma.

### Medulloblastoma patients’ demographic data, clinical presentation, and surgery

The demographic and treatment characteristics of the 41 confirmed MB patients were analyzed. The median age at diagnosis was 6 years old (range, 0.25–16 years). A male preponderance was observed with a male-to-female ratio of 2.4: 1. There were 31 children aged ≥3 years old and ten infants (<3 years old). The most common clinical presentations were headache (63.4%), nausea/vomiting (63.4%), unsteady gait (48.8%), and cerebellar dysfunction symptoms/signs (43.9%). The pre-diagnostic symptom interval (PSI) duration varied from 1 week to 16 weeks, and the median duration of PSI was 3 weeks. Data on PSI was unavailable in eight patients. Sixteen patients (39%) had upfront gross total resection (GTR), and another three patients achieved complete resection after second-look surgery. Twelve patients (29%) had near-total resection (NTR) in which three of them underwent second-look surgery to achieve NTR. Radiological subtotal resection (STR) was observed in nine patients (22%) and four of them had second-look surgery. The extent of surgical resection information was missing in one patient (**Table 1**). A ventriculoperitoneal shunt (VP) was inserted in 24 patients (58.5%). Overall, eight families refused treatment and the abandonment rate for the whole cohort was 19.5%.

### Children ≥ 3 years old with medulloblastoma

#### Medulloblastoma histological variants, molecular subgroup, and risk stratification

Based on histological reports, seven (22.6%) patients had classic histology, DN was reported in three (9.7%) patients and in 21 (67.7%) patients the histological variant was not specified. By methylation, four patients were classified as WNT (12.9%), SHH was identified in four patients (12.9%), G3 in seven patients (22.6%), and 16 patients were stratified as G4 MB (51.6%). Two G3 patients had *MYCC* amplification. For G4, one patient was diagnosed to have *MYCC* and another two patients were found to have *MYCN* amplification with 850k DNA methylation array technique. Radiological imaging and cerebrospinal fluids analysis revealed metastatic disease in 11 patients, 19 patients had localized disease and data was missing in one patient. Sixteen patients (51.6%) were stratified as HR and the remaining 15 patients were stratified as SR (**Table 1**). The abandonment rate was 22.6% (seven patients) in older children; five HR patients refused radiotherapy post-surgery and two SR patients defaulted after surgery and radiation.

#### Treatment characteristics and relapse pattern

**Tables 1**, **2** summarize the treatment and relapse patterns. Twenty-five patients (SR MB, n=14, HR MB, n=11) were given up-front radiation with a median interval between surgery and initiation of radiotherapy of 41 days (18-152 days). Six patients (SR MB, n=1, HR MB, n=5) did not receive radiotherapy due to poor neurological status and family refusal.

**Table 2:** Outcome of relapsed medulloblastoma patients.

Sub groups	Mt	Initial Diagnosis	Site of tumor at diagnosis	Site of recurrence/progression	TTR/P from the last day of treatment	Surgery during recurrence	Histology during recurrence	Diagnosis at recurrence	MRI spine at recurrence/progression	CSF cytology	Salvage treatment during recurrence/progression (No. courses)	Outcome
**^WNT^**	**Not done**	**NOS MB, WNT**	**PF**	**PF**	**4 months (after surgery, refused CTX/RT)**	**GTR**	**NOS MB**	**MB**	**Neg**	**NP**	**Palliative support**	**Dead**
**^SHH^**	**3**	**Classic MB, SHH-INF**	**PF**	*PF	**Recurrence** **(during CTX)**	**NP**	**NA**	**MB**	**NP**	**NP**	**Palliative support**	**Dead**
	**0**	**NOS MB, SHH-INF type 2**	**PF**	**PF**	**18 months from EOT**	**GTR**	**NOS MB**	**MB**	**Neg**	**Neg**	**CSI 36Gy and PF 54Gy with 8 courses of CCNU, Cis, VCR**	**Alive**
	**0**	**NOS MB, SHH AD type 3**	**PF**	**PF**	**Recurrence** **(during CTX)**	**NP**	**NA**	**MB**	**Neg**	**Neg**	**SRS 15Gy**	**Alive**
	**0**	**NOS MB, SHH AD type 3**	**PF**	**PF**	**2.5 months** **from EOT**	**PR**	**NOS MB**	**MB**	**Neg**	**Neg**	**Palliative support**	**Dead**
	**0**	**NOS MB, SHH-INF type 1**	**PF**	**PF**	**PD (during CTX)**	**No**	**No**	**MB**	**Neg**	**NP**	**Palliative support**	**Dead**
**G3**	**3**	**Classic MB, G3,** **c-myc**	**PF with spinal metastasis**	**PF with spinal metastasis**	**3 months** **from EOT**	**STR**	**Classic MB, G3,** **c-myc**	**MB**	**Metastasis**	**Neg**	**CSI 35Gy and PF 54Gy**	**Dead**
	**3**	**NOS MB, G3, c-myc**	**PF with spinal metastasis**	**PF**	**PD (Refused RT, defaulted CTX)**	**NP**	**NA**	**MB**	**Metastasis**	**NP**	**Palliative support**	**Dead**
	**0**	**Classic MB, G3,** **c-myc**	**PF**	**Right frontal lobe**	**12 months** **from EOT**	**Biopsy**	**MB Classic**	**MB**	**Neg**	**Neg**	**Focal re-irradiation 54Gy** **Oral etoposide for 1 month**	**Dead**
	**0**	**Classic MB, G3,** **c-myc**	**PF**	**PF, intracranial leptomeningeal & spine**	**PD (during CTX)**	**NP**	**NA**	**MB**	**Metastasis**	**NP**	**Palliative support**	**Dead**
**G4**	**1**	**Classic MB, G4**	**PF**	**PF**	**4 months** **from EOT**	**NP**	**NA**	**MB**	**Neg**	**NP**	**Palliative support**	**Dead**
	**0**	**Desmoplas-tic MB,G4**	**PF**	*PF	**37.5 months** **from EOT**	**NP**	**NA**	**MB**	**Not done**	**NP**	**Palliative support**	**Dead**
	**0**	**Classic MB, G4**	**PF**	**PF with spinal metastasis**	**30 months (after RT, refused CTX)**	**NP**	**NA**	**MB**	**Metastasis**	**NP**	**Palliative support**	**Dead**
	**3**	**NOS MB, G4, c-myc**	**PF with spinal metastasis**	*Third ventricle	**Recurrence (during CTX)**	**NP**	**NA**	**MB**	**NP**	**NP**	**Palliative support**	**Dead**
	**0**	**NOS MB, G4**	**PF**	**PF and spinal metastasis**	**26 months** **from EOT**	**NP**	**NA**	**MB**	**Metastasis**	**NP**	**Palliative support**	**Dead**

AD, adult; CCNU, lomustine; Cis, cisplatin; CTX, chemotherapy; CSI, craniospinal radiotherapy; EOT, end of treatment; G3, Group 3; G4, Group 4; INF, infant; MB, medulloblastoma; Mt, metastasis during initial diagnosis; NA, not applicable; NOS, non-otherwise specified; NP, not performed; PD, progressive disease; PF, posterior fossa; RT, radiotherapy; SHH, sonic hedgehog; SRS, stereotactic radiosurgery; STR, subtotal resection; TTR/P, time to relapse/progression; VCR, vincristine; WNT, Wingless.

*MRI spine was not done at recurrence/progression.

Color shading signifies certain molecular subgroup in medulloblastoma.

#### Clinical course for *standard*-risk MB patients (n=15)

Regarding radiotherapy, eleven patients were treated with a higher CSI dose of 36Gy and primary site boost (PSB) of 54Gy according to physicians’ discretion due to difficulties in obtaining magnetic resonance imaging (MRI) of the spine and CSF cytology within the recommended interval for accurate disease staging. In addition, CSF cytology results were unreliable due to technical difficulties in transportation, storage, and interpretation. All but one of these patients (10/11) received radiotherapy within 49 days of surgery, with concurrent weekly vincristine. One patient received radiotherapy at 54 days from surgery due to post-operative infection. Only two patients received a standard CSI dose of 23.4Gy with PSB of 54-56Gy within 49 days of surgery. One G4 patient died from an *Acinetobacter Baumanii* VP shunt infection during radiotherapy. One patient with G3 MB was palliated by the treating physician due to significant neurological impairment post-surgery. Of 13 patients treated with radiation without interruption, nine patients eventually completed eight courses of the A9961 chemotherapy regimen (7), whilst three patients received incomplete courses of adjuvant chemotherapy, and one patient’s parents refused adjuvant chemotherapy. Neutropenic sepsis, treatment abandonment, and disease recurrence were the contributing factors to receiving incomplete chemotherapy in the three patients. Out of the nine patients who completed full treatment, six were still in remission at the last follow-up. However, the remaining three patients died due to combined, distant, and local recurrence at 26, 12, and 37.5 months respectively after completing initial treatment. Of these, one patient had G3 MB with *MYCC* amplification, and another two patients had G4 MB. They were referred to the palliative team for the continuation of end-of-life care management. One G4 MB patient who refused adjuvant chemotherapy had primary recurrence with spinal metastasis 30 months following the completion of radiation therapy. He was not salvaged following recurrence (**Tables 1**, **2**).

#### Clinical course for high-risk MB patients (n=16)

**Tables 1**, **2** summarize the treatment and relapse patterns. Five families refused radiotherapy. Of these, one G3 MB patient with *MYCC* amplification abandoned the treatment after one course of Head Start II (HS II) chemotherapy and the patient passed away with primary and spinal disease progression (12). The remaining four patients’ parents refused treatment after surgery and all these patients died of progressive disease. The remaining 11 patients received CSI at a dose of 36-45Gy with a PSB of 54Gy. Four of these patients received delayed radiotherapy on day 56, day 59, day 77, and day 152 post-surgery due to a limited number of linear accelerators, lack of anesthetists to provide sedation during radiation, and parental phobia of radiotherapy. These patients did not receive chemotherapy as a bridging therapy after surgery while waiting for radiotherapy commencement. Of the 11 patients, ten patients were treated with weekly vincristine during radiotherapy, followed by A9961 chemotherapy (7). One patient received chemotherapy as per the Pediatric Oncology Group (POG) 9031 regimen (9) but without concurrent chemotherapy during radiation. Among those who received delayed radiation, one G4 patient with *MYCC* amplification treated with radiotherapy on day 77 post-surgery had a distant recurrence at the third ventricle after the first course of chemotherapy and died without salvage treatment. Another G4 MB patient with delayed radiotherapy on day 152 post-surgery had primary site recurrence after 4 months of treatment and received palliative care (**Tables 1**, **2**). The remaining two patients with delayed radiotherapy were still in complete remission during the last follow-up.

#### Survival outcomes

**Figure 1:**
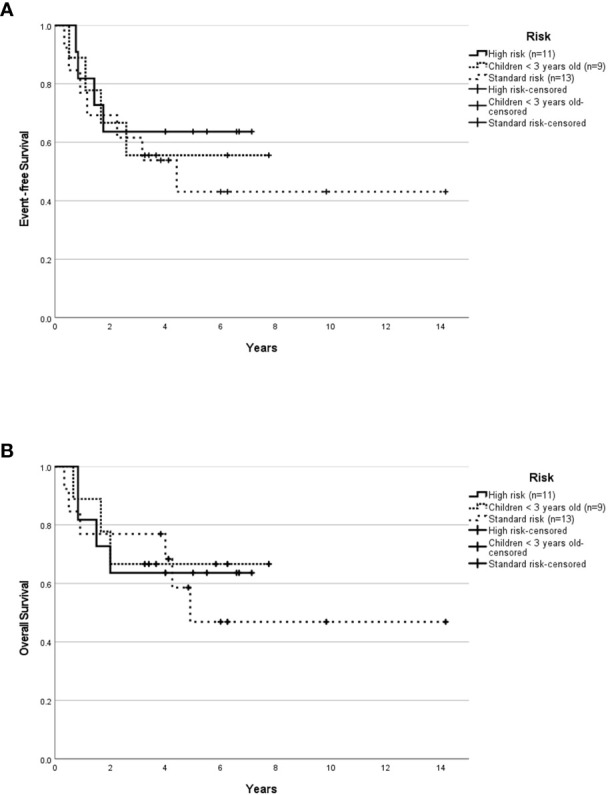
Survival outcome of childhood medulloblastoma (excluding the abandonment) based on risk stratification. Event-free survival **(A)** and overall survival **(B)** for the whole cohort according to risk stratification after excluding the abandonment.

**Figure 2:**
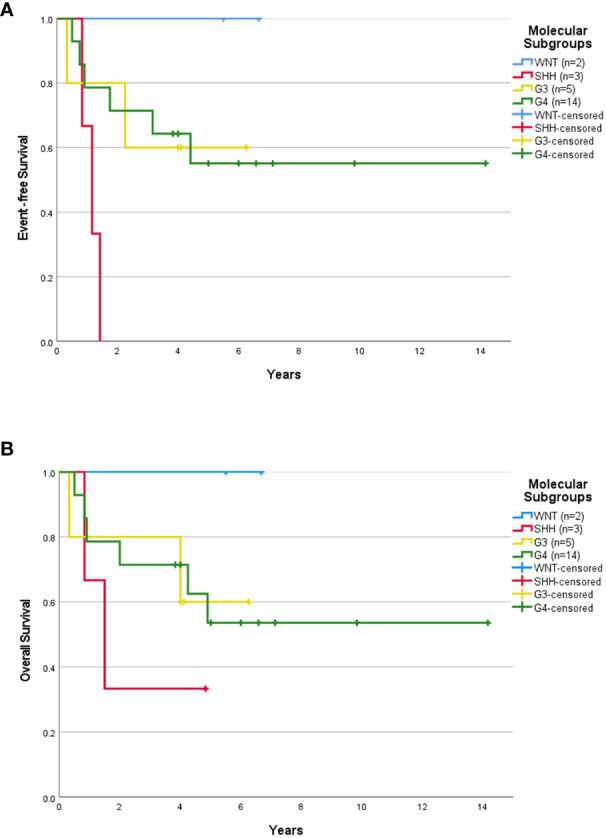
Survival outcome of childhood medulloblastoma > 3 years old (excluding the abandonment) based on molecular subgroups. Event-free survival **(A)** and overall survival **(B)** for children > 3 years old according to molecular subgroups after excluding the abandonment.

Median follow-up for children ≥3 years old was 4.0 years (range, 0.04-14.16 years). The 5-year EFS rates for SR and HR patients were 37.3 ± 13.3% and 43.8 ± 12.4% respectively. The 5-year OS rates for SR and HR were 40.6 ± 14.1% and 43.8 ± 12.4% respectively. The 5-year EFS rates for non-metastatic and metastatic patients were 35.1 ± 11.4% and 54.5 ± 15.0%. Survival based on molecular subgroups was undertaken only for G3 and G4 patients, as there were too few WNT and SHH patients to generate survival curves. 5-year EFS and OS rates were 42.9 ± 18.7% for G3 respectively. Whilst, the 5-year EFS and OS rates for G4 were 48.2 ± 13.6% and 46.9 ± 13.2% respectively. Of the four WNT patients, all were classified as high-risk based on the presence of either residual tumor >1.5cm^2^ and/or metastatic disease. Two of these WNT patients received 36Gy CSI followed by eight cycles of A9961 chemotherapy and are alive disease-free 6 and 5 years from the diagnosis. Treatment was abandoned after a GTR in one WNT patient, who developed a local relapse four months later and died. In another WNT patient, treatment was abandoned after an STR, and the patient was lost to follow-up. Of the four SHH patients, one patient relapsed locally during treatment, was salvaged with focal stereotactic radiosurgery of 15Gy, and remained in remission. Another SHH patient relapsed locally after 2.5 months of treatment and succumbed due to disease progression. The third SHH patient abandoned the treatment after four courses of chemotherapy and was lost to follow-up. Another metastatic SHH patient developed disease recurrence while on treatment and died due to disease progression. After censoring those patients where treatment was abandoned, the 5-year EFS rates for SR and HR were 43.1 ± 14.7% and 63.6 ± 14.5% respectively (**Figure 1A**). The 5-year OS rates for SR and HR were 46.9 ± 15.6% and 63.6 ± 14.5% respectively (**Figure 1B**). The 5-year EFS rates for non-metastatic and metastatic patients were 44.4 ± 13.5% and 66.7 ± 15.7%. According to molecular subgroups, G3 MB, 5-year EFS, and OS were both 60 ± 21.9%. G4 MB showed 5-year EFS and OS rates of 55.1 ± 13.9% and 53.6 ± 14.2% respectively (**Figures 2A, B**).

### Children <3 years old with medulloblastoma (n=10)

#### Medulloblastoma histology subclass, molecular subgroup, and risk stratification

Three (30%) patients had classic histology, two (20%) patients had MBEN subclass, DN was reported in three (30%) patients and histological variant was not specified in two (20%) patients. By methylation, seven patients were classified as SHH subgroups (70%), G3 MB was seen in three patients (30%) and two of these had *MYCC* amplification. Eight patients had localized disease, one patient presented with metastatic disease and staging data was missing in one patient (**Table 1**).

#### Treatment characteristics and relapse pattern

Of the ten patients, for one SHH patient, the family declined treatment following surgical resection, whilst the remainder (90%) were treated according to radiotherapy-sparing regimens (n=4 [HS II], n=3 [POG Baby Brain], n=2 [ACNS 1221]) (12, 23, 24). One patient with G3 and *MYCC* amplification had a primary and distant relapse 3 months into treatment. This patient received salvage treatment with CSI of 35Gy and primary tumor boost of 54Gy but succumbed due to disease progression. POG Baby Brain protocol was administered in three patients (two SHH and one G3 with *MYCC* amplification) and all of them had disease progression (24). Of these, one patient with SHH MB was salvaged with a CSI of 36Gy and a primary tumor boost of 54Gy followed by the A9961 regimen and is still in remission 5.83 years from completion of treatment (**Tables 1**, **2**) but the other two patients went on to receive palliative therapy. The extent of surgical resection did not appear to influence the outcome in young children. Overall, the abandonment rate was 10% (one patient) in younger children.

#### Survival outcomes

**Figure 3:**
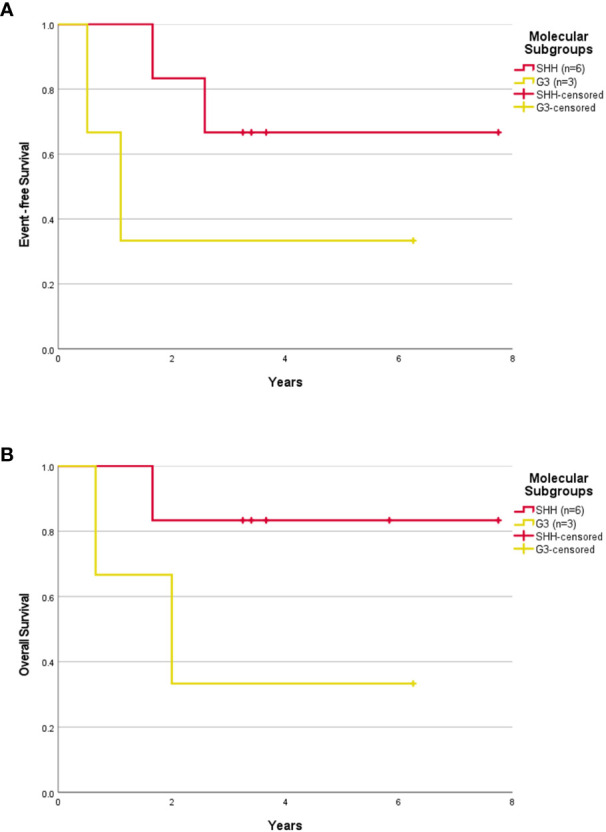
Survival outcome of childhood medulloblastoma ≤ 3 years old (excluding the abandonment) based on molecular subgroups. Event-free survival **(A)** and overall survival **(B)** for children > 3 years old according to molecular subgroups after excluding the abandonment.

The median follow-up was 3.32 years (range, 0.33-7.75 years). The 5-year EFS and OS rates were 50.0 ± 15.8% and 60.0 ± 15.5% respectively. The 5-year EFS and OS rates for SHH patients were 57.1 ± 18.7% and 71.4 ± 17.1% respectively. After censoring the patient who abandoned treatment, the 5-year EFS and OS rates were 55.6 ± 16.6% and 66.7 ± 15.7% respectively for the whole cohort, and 66.7 ± 19.2% and 83.3 ± 15.2% respectively for the SHH group (**Figures 1A, B**, **3A, B**). Numbers were too small to generate survival curves for G3 patients. Of the three G3 patients, two died of progressive disease and both had *MYCC* amplification. The other remains in remission 6.25 years following treatment with HSII.

## Discussion

The present study is the first study in Malaysia that reports the molecular subgrouping in childhood MB with clinical descriptions. With the rapid advancement in molecular profiling and incorporation of molecular information into clinical risk stratification, the management of childhood MB has undergone a paradigm shift. However, implementing these strategies in daily practice is challenging for most centers in LMIC due to a lack of expertise and associated high costs. IHC has formed an integral component of histopathological diagnosis for decades, however several reports have shown significant inter-observer variability in the histopathological diagnosis of many CNS tumors (25–27). Capper et al. reported that 129 out of 1104 (12%) CNS tumor cases had discordant histopathological diagnosis based on DNA methylation which resulted in the revision of the original histopathological diagnosis in favor of the DNA methylation classification (19). Consistent with this finding in our study a similar discordant rate of 15% was also observed between local histopathological diagnosis and DNA methylation profiling. The critical importance of an accurate diagnosis in assigning the most appropriate treatment is evident in our series. Patients with GBM, Ewing sarcoma, and MPNST-like sarcoma received more intensive treatment regimens, including craniospinal radiotherapy (CSI) of 36Gy with PSB of 54Gy and intensive chemotherapy than the respective standard of care therapies. This may have resulted in prolonged hospitalization with additional morbidity to the patient and the added socio-economic burden to the family. In the absence of dedicated neuropathologists in many LMICs, DNA methylation would represent an ideal tool for accurate diagnosis, if the costs were not the main limiting factor for its implementation.

The current WHO CNS tumor classification identifies four histopathological subclasses of MB; classic, DN, LCA, and MBEN (28). In our series, tumor histological variants were only reported in 44% of MB patients, highlighting the limited neuropathology expertise that exists in LMIC. Several studies have reported that young children with DN/MBEN subtype showed an excellent outcome, whilst LCA histology demonstrated a dismal prognosis (29, 30). Previous studies in LMIC have used simpler techniques, such as fluorescence *in situ* hybridization (FISH), and specific IHC markers as surrogate methods, to molecularly subgroup MB. GAB1, YAP1, filamen A along with beta-catenin IHC antibodies are used to classify MB into WNT, SHH, and non-WNT/SHH subgroups (31). These techniques are easily applicable and cost-effective (20, 21). For example, specific IHC with positive nuclear beta-catenin and FISH demonstrating monosomy 6 can be used to identify WNT tumors. However, caution has been advised in making a diagnosis of WNT tumors using either nuclear beta-catenin alone as false positives occur or monosomy 6 alone as this marker has been occasionally observed in other subgroups (22, 32, 33). In addition, beta-catenin IHC alone may lead to an incorrect diagnosis of a WNT subgroup due to difficulty in interpreting patchy nuclear accumulation in some tumors (15, 32). Moreover, these specific IHC antibodies are unable to differentiate G3 and G4 tumors. This highlights the importance of DNA methylation profiling method which has a substantial impact on diagnostic precision in CNS tumors across the globe. Hence, DNA methylation testing has become an internationally accepted method for accurate molecular identification (19) and has been included in the recently revised fifth edition of the WHO classification of CNS tumors (WHO CNS 5^th^ edition) (28).

MRI is the preferred first-line modality in MB, and traditionally, it has been used for diagnosis, surgical guidance, staging, treatment response evaluation, and surveillance during follow-up. However, recent studies have shown encouraging data regarding radiogenomics features of MB with distinct imaging characteristics (radio-phenotypes) correlating with specific molecular subgroups (molecular phenotypes) (34). It is increasingly recognized that imaging features of MB can reflect the underlying disease biology, which may serve as a helpful tool to predict the molecular subgroups of MB, especially in LMIC (35). Even though there were no specific pathognomonic features for each molecular subgroup, some radiological characteristics were more peculiar and predominant in one subgroup than others (34–38). More work needs to be done to validate these correlations that would benefit clinicians who do not have access to DNA methylation investigation.

For children ≥ 3 years of age, the proportion of WNT, SHH, and G3 patients were consistent with high-income countries (HIC) (WNT 12.9% versus 9-17.4%; SHH 12.9% versus 15%; G3 22.6% versus 21.3-32%) but the proportion was higher for G4 (51.6% versus 44-45.6%) (16, 17). For children <3 years of age, the relative proportions of patients in each of the four molecular subgroups was in keeping when compared with HIC, with a majority of SHH (70% versus 65%), 30% G3 patients and no WNT patients (34, 35). However, in contrast to other studies revealing approximately less than 10% G4 patients, in our cohort there were no G4 patients (29, 30, 39, 40). The age cutoff for infants and young children varies from one cooperative group to another. There is no consensus on the age cutoff for infants and young children with MB. Some infant studies include children up to 3 years old, while others extend the age cutoff to 4 or 5 years old. Hence, the differences in age cutoff in MB treatment protocols exhibit the variances in the proportion of G4 MB in young children. Furthermore, the median age in our study was 6 years old, and G4 MB was most frequently seen in older children. These could be the reasons for the higher proportion of G4 MB in older children and the absence of G4 MB among children < 3 years old in this study, in addition to the racial differences and small sample size. All five patients with *MYCC* amplification in G3 (n=4) and G4 (n=1) passed away with disease progression, whilst patients with *MYCN* amplification in G4 (n=2) were still in remission during the last follow-up. This result is consistent with the SJMB03 trial report where *MYCC* amplification was associated with inferior survival whereas *MYCN* amplification was not associated with G3 and G4 MB outcomes (17).

For children ≥ 3 years of age with HR MB, survival outcomes were comparable with reports from developed countries, after removing patients where therapy was abandoned. In sharp contrast, the survival for patients with SR MB was dismal (5-year OS 43.1%) despite receiving 36Gy CSI. This was likely due to treatment-related complications such as sepsis and post-surgical mortality. Of note, the 5-year OS outcomes in older patients with G4 MB were inferior when compared to developed countries even after censoring the abandonment cases (53.6% versus 77-95%) (6, 8, 17). The reason for this finding in part is likely related to toxic deaths. The WNT subgroup has been shown to have an excellent outcome, even for the small proportion of patients with high-risk features (6, 8, 17). Consistent with this, both WNT MB patients in our series, who were treated using HR therapy based on the Chang staging system, survived despite having metastatic disease and residual tumor >1.5cm^2^. Hence, molecular classification information is important for treatment strategy and disease prognostication. In addition, two G4 patients presented at the age of 3 years, they received upfront radiation and are long-term survivors. This is an important issue in LMIC as radiation in young children is associated with significant neurocognitive deficits when early intervention programs and special education resources are very limited in the community (22).

The 5-year EFS and OS outcomes for children <3 years of age in our cohort were more in keeping with survival from developed countries (12). Young children with SHH clearly had a better outcome than older children in our study consistent with previous reports (28, 39). Using the SJYC07 treatment regimen, Robinson et al. reported a superior outcome for the SHH-II subtype compared with the SHH-I subtype (39). However, the addition of intraventricular methotrexate appears to negate the inferior outcome associated with SHH-I subtype (29). Given the small patient numbers, we did not further analyze the survival outcome for SHH subtypes (SHH-1 and SHH-II). As noted by others, G3 MB did much worse due to the frequent presence of *MYCC* amplification (29, 40). Moreover, aggressive surgical intervention might not be indicated in young children with MBEN and DN histology as the presence of residual tumor was not associated with the dismal outcome in our cohort. MBEN and DN histology variants are known to have excellent outcomes (29, 30).

Treatment abandonment due to cultural beliefs that traditional medicine is superior, lack of awareness regarding childhood cancer trajectory among parents, ideas that cancer is incurable, low socioeconomic status, poor parental education level, long travel time with lack of housing facilities for families from remote areas, painful procedures, and treatment adverse effects and toxicity were well-recognized contributing factors to inferior outcomes (41, 42). The overall abandonment rate in our study was 19.5%. A single-center study on challenges treating pediatric MB in Malaysia reported a treatment dropout rate of 35.3% (42). Similarly, the abandonment rates of MB in other developing countries from Asia ranged from 31% to 36.4% (43–45). In contrast, the treatment refusal rates were only between 0.6% to 5.7% in HIC (6, 17, 39). Therefore, identifying the risk factors and prioritizing strategies to reduce the incidence of treatment rejection is crucial in LMIC to close the survival gap. Optimal care can be achieved by providing free lodging and food to patients and families, financial support for travel, social support, efficient communication with detailed and repeated counseling, and effective procedural sedation and analgesia. Notably, developing satellite cancer centers for patients living in rural areas and initiating a contact tracing mechanism for defaulters would certainly contribute to mitigating some aspects of treatment abandonments (46). Additionally, organizing regular national campaigns may cultivate health-seeking behavior by creating public awareness about the curability of cancer and its early warning signs (46). Importantly, our study’s treatment-related complications, such as septicemic death and post-surgical mortality, were concerning. The critical factors for the dismal outcome were the lack of specialized pediatric neuro-oncology multidisciplinary services, limited human resources and infrastructure, poor supportive care, and deficiency in the internal health delivery system (47). In our cohort, radiotherapy was also delayed in several patients due to a limited number of linear accelerators, frequent machine breakdowns, and a lack of staff to provide sedation or general anesthesia, which resulted in a long waiting list (42). In addition, late parental consent for treatment and post-operative complications contributed to delayed radiotherapy. These factors caused significant barriers to commencing radiotherapy on time, leading to poor adherence to treatment guidelines (42). Hence, building human resource capacity through structured national education and training programs is essential to increase the number of skilled and experienced pediatric neuro-oncology multidisciplinary healthcare professionals to improve the service quality and diagnostic capacity to avoid delays in diagnosis, misdiagnosis, and mistreatment. Furthermore, increasing focus on healthcare financing for catastrophic illness, especially allocating adequate budget, supporting human resource training, establishing specialized diagnostic and treatment cancer centers for childhood CNS tumors, improving the availability of novel drugs and supplies, providing equipment such as radiotherapy and radiology machines, and periodic monitoring of cancer registry should be the priority (46, 47).

This study is the first study reporting on the four molecular subgroups of MB among children in Malaysia. The study’s main limitation was that it was a retrospective study with a relatively small sample size. The challenges of small sample size were augmented after patients were divided into four molecular subgroups. Additionally, missing patients’ records and incomplete clinical and pathological data limit the analysis and interpretation of the study. Data regarding radiogenomics features of MB to determine the correlation between imaging characteristics and molecular subgroups of MB were not collected for analysis.

In conclusion, the discrepancy between histological diagnoses and DNA methylation profiling highlights the importance of DNA methylation profiling in improving the accuracy of diagnosis. OS for children ≥3 years of age with HR MB was consistent with other reports. However, OS was very poor for those classified with SR. Most infants had SHH MB, and their EFS and OS were comparable to those reported in high-income countries. Due to the relatively small patient cohort and the high treatment abandonment rate with treatment-related mortality, definite conclusions regarding the prognostic significance of the four molecular subgroups of MB cannot be made for children aged ≥ 3 years. Implementing this high-technology investigation would assist pathologists in improving the diagnosis and provide molecular subgrouping of MB as we move toward subgroup-specific therapies. However, treatment abandonment, delayed radiotherapy, and treatment-related complications are the priorities that need to be addressed to maximize the benefits of such technology.

## Data availability statement

The original contributions presented in the study are included in the article/supplementary material, further inquiries can be directed to the corresponding author/s.

## Ethics statement

The studies involving humans samples were approved by Ministry of Health (MOH) Medical Research and Ethics Committee (NMRR-17-991-35677) and University Malaya Medical Centre Medical Research Ethics Committee (MREC-2016112-4485). The studies were conducted in accordance with the local legislation and institutional requirements. The human samples used in this study were acquired from a by- product of routine care or industry. Written informed consent for participation was not required from the participants or the participants’ legal guardians/next of kin in accordance with the national legislation and institutional requirements.

## Author contributions

RR: Conceptualization, Formal Analysis, Methodology, Writing – original draft, Writing – review & editing. AJT: Data curation, Writing – review & editing. VJ: Data curation, Writing – review & editing. OW: Investigation, Writing – review & editing. HM: Investigation, Writing – review & editing. NHAR: Investigation, Writing – review & editing. TY: Data curation, Writing – review & editing. KG: Data curation, Writing – review & editing. AT: Data curation, Writing – review & editing. SY: Data curation, Writing – review & editing. GO: Data curation, Writing – review & editing. HA: Data curation, Writing – review & editing. DJ: Formal Analysis, Investigation, Writing – review & editing. EB: Conceptualization, Supervision, Writing – original draft, Writing – review & editing. NG: Conceptualization, Formal Analysis, Methodology, Supervision, Writing – original draft, Writing – review & editing.
